# VSV-Based Vaccines Reduce Virus Shedding and Viral Load in Hamsters Infected with SARS-CoV-2 Variants of Concern

**DOI:** 10.3390/vaccines10030435

**Published:** 2022-03-12

**Authors:** Kyle L. O’Donnell, Tylisha Gourdine, Paige Fletcher, Kyle Shifflett, Wakako Furuyama, Chad S. Clancy, Andrea Marzi

**Affiliations:** 1 Laboratory of Virology, Division of Intramural Research, National Institute of Allergy and Infectious Diseases, National Institutes of Health, Hamilton, MT 59840, USAtylisha.gourdine@nih.gov (T.G.); paige.fletcher@nih.gov (P.F.); fish.shifflett@gmail.com (K.S.); wfuruyama@nagasaki-u.ac.jp (W.F.); 2 Rocky Mountain Veterinary Branch, Division of Intramural Research, National Institute of Allergy and Infectious Diseases, National Institutes of Health, Hamilton, MT 59840, USA; chad.clancy@nih.gov

**Keywords:** severe acute respiratory syndrome coronavirus-2, COVID-19, vesicular stomatitis virus, intranasal vaccination

## Abstract

The continued progression of the COVID-19 pandemic can partly be attributed to the ability of SARS-CoV-2 to mutate and introduce new viral variants. Some of these variants with the potential to spread quickly and conquer the globe are termed variants of concern (VOC). The existing vaccines implemented on a global scale are based on the ancestral strain, which has resulted in increased numbers of breakthrough infections as these VOC have emerged. It is imperative to show protection against VOC infection with newly developed vaccines. Previously, we evaluated two vesicular stomatitis virus (VSV)-based vaccines expressing the SARS-CoV-2 spike protein alone (VSV-SARS2) or in combination with the Ebola virus glycoprotein (VSV-SARS2-EBOV) and demonstrated their fast-acting potential. Here, we prolonged the time to challenge; we vaccinated hamsters intranasally (IN) or intramuscularly 28 days prior to infection with three SARS-CoV-2 VOC—the Alpha, Beta, and Delta variants. IN vaccination with either the VSV-SARS2 or VSV-SARS2-EBOV resulted in the highest protective efficacy as demonstrated by decreased virus shedding and lung viral load of vaccinated hamsters. Histopathologic analysis of the lungs revealed the least amount of lung damage in the IN-vaccinated animals regardless of the challenge virus. This data demonstrates the ability of a VSV-based vaccine to not only protect from disease caused by SARS-CoV-2 VOC but also reduce viral shedding.

## 1. Introduction

Early during the COVID-19 pandemic, caused by severe acute respiratory syndrome coronavirus 2 (SARS-CoV-2), it became apparent that greater challenges lay ahead in comparison to the outbreaks caused by SARS-CoV and MERS-CoV in 2002 and 2012, respectively. SARS-CoV-2 can spread effectively from people that are pre-symptomatic or asymptomatic, making surveillance and epidemiology challenging [1]. Due to the rapid global dissemination of the virus, research and countermeasure development were conducted on an unprecedented scale and at an unprecedented speed. As the virus continued to spread, it accumulated mutations, leading to newly emerging viral variants circumventing the growing resistance the population was acquiring through vaccination and natural infection [2,3,4,5]. One of the emerging SARS-CoV-2 variants of concern (VOC), the Alpha variant, had acquired 23 mutations within the spike (S) protein to increase binding affinity to the ACE2 receptor and undergone a deletion of amino acids (aa) 69 and 70 that has been associated with increased viral escape in immunocompromised individuals [6]. The Beta variant harbors similar S protein mutations in addition to N501Y, E484K, and deletion of aa 242–244 that increase binding affinity to host cells. This increased binding affinity may decrease the efficacy of existing vaccines [7,8,9]. The Delta variant harbors 10 unique mutations in the S protein in comparison to prior VOC. Of note, the L452R and T478K mutations as well as the deletion of aa 156–157 are located in key antigenic regions of the receptor-binding domain and N-terminal domain. Another mutation, P681R, is close to the furin cleavage site and allows for increased infectivity [10]. The emergence of VOC is driven by the need to evade acquired immunity and increase transmissibility. As a result, the key question is whether existing vaccine-induced immunity combats emerging VOC threats. At this point, approximately half of the world’s population has been fully vaccinated against SARS-CoV-2. However, durability projections of this immunity are still unclear [11]. Through mass vaccination against COVID-19, the effectiveness to protect against severe disease in a real-world setting is unquestioned [2,12,13]. However, decreases in immunologic function and increases of breakthrough infection point toward waning vaccine-induced immunity over time [2,14,15,16]. A systematic review by Zhang et al. determined that breakthrough cases rose from 1% of fully vaccinated people prior to the emergence of the Delta variant to 35% when it became the dominant circulating virus. Only 37% of the patients that met the criteria for being critically ill during hospitalization were fully vaccinated [17]. Waning vaccine efficacy over time likely contributed to the increased percentage of breakthrough cases [18]. In order to lower the numbers of breakthrough cases, the CDC encouraged booster vaccinations in the USA to increase the magnitude of the immune response to retain vaccine efficacy. With the encouragement of booster vaccinations, further research into novel vaccine constructs to support the demand for booster doses has been supported. Viral vector vaccines, such as the AstraZeneca ChAdOx1 nCOV/AZD1222 vaccine and the NDV-HXP-S, have shown promising results in preclinical studies and clinical application against VOC [19,20,21,22]. We sought to utilize the vesicular stomatitis virus (VSV)-based vaccination platform to construct a monovalent and bivalent vaccine against SARS-CoV-2. 

The advantages of the VSV platform include the ease of genetic manipulation to incorporate new antigens, a short time to immunity, and the ability to produce high titer stocks for the production of vaccine doses quickly [23,24]. A major concern with viral vector platforms is the risk of preexisting immunity circumventing the protective efficacy of the vaccine. The seroprevalence of VSV in the general population is extremely low, and preexisting immunity is directed against the VSV glycoprotein (GP) which has been removed from our constructs [25]. In addition to circumventing limited preexisting immunity, the removal of the VSV GP bolsters the safety profile of our vaccine constructs, as wild-type VSV infection is lethal in hamsters [26,27]. Several routes of vaccination, including intramuscular (IM) and intranasal (IN) routes, have demonstrated VSV-based vaccine efficacy against various viral pathogens, including Ebola virus (EBOV), Marburg virus, Lassa virus, Andes virus, Zika virus, and highly pathogenic avian influenza virus in animal models [23,28,29,30,31,32]. We have previously demonstrated the fast-acting potential of VSV-based SARS-CoV-2 vaccines in hamsters [33] and nonhuman primates (NHPs) [34]. In this study we sought to determine whether a 28-day vaccination to challenge schedule would confirm vaccine efficacy against multiple VOC. In addition, we investigated whether the observed differences by routes of vaccine administration were retained or abrogated with this longer time course. We demonstrate that the optimal route of vaccination is IN administration, resulting in protective efficacy against multiple VOC compared to IM vaccination in the Syrian golden hamster model. 

## 2. Materials and Methods

### 2.1. Ethics Statement

All infectious work with SARS-CoV-2 was performed in the high-containment laboratories at the Rocky Mountain Laboratories (RML), Division of Intramural Research, National Institute of Allergy and Infectious Diseases, National Institutes of Health. RML is an institution accredited by the Association for Assessment and Accreditation of Laboratory Animal Care International (AAALAC). All procedures followed standard operating procedures (SOPs) approved by the RML Institutional Biosafety Committee (IBC). Animal work was performed in strict accordance with the recommendations described in the Guide for the Care and Use of Laboratory Animals of the National Institute of Health, the Office of Animal Welfare and the Animal Welfare Act, United States Department of Agriculture. The studies were approved by the RML Animal Care and Use Committee (ACUC). Procedures were conducted on animals anesthetized by trained personnel. All efforts were made to ameliorate animal welfare and minimize animal suffering; food and water were available *ad libitum*.

### 2.2. Animal Study

Seventy-two Syrian golden hamsters (5–8 weeks of age, female) were obtained from Envigo (Indianapolis, IN, USA) and used in this study. The hamsters were randomly selected into groups of 6. On the day of vaccination, hamsters received a single dose of 1 × 10^5^ PFU of VSV-SARS2 or VSV-SARS2-EBOV by the IM (thigh) or IN route. Control animals received the same dose of a control vaccine (VSV-EBOV) by either the IM (*n* = 3 per VOC) or IN (*n* = 3 per VOC) route; samples were combined and analyzed as one control group (*n* = 6). On day 0, all hamsters were challenged IN with 1 × 10^5^ TCID_50_ SARS-CoV-2, as previously described [35,36]. At 4 days post-challenge (DPC), all animals were euthanized for sample collection.

### 2.3. Cells and Viruses

VeroE6 cells were grown at 37 °C and 5% CO_2_ in Dulbecco’s Modified Eagle’s Medium (DMEM) (Sigma-Aldrich, St. Louis, MO, USA) containing 10% fetal bovine serum (FBS) (Wisent Inc., St. Bruno, QC, Canada), 2 mM L-glutamine (Thermo Fisher Scientific, Waltham, MA, USA), 50 U/mL penicillin (Thermo Fisher Scientific), and 50 μg/mL streptomycin (Thermo Fisher Scientific). SARS-CoV-2 Alpha isolate (hCOV_19/England/204820464/2020) [36], SARS-CoV-2 Beta isolate (hCoV-19/South African/KRISP-K005325/2020) [36], or SARS-CoV-2 Delta isolate (hCoV-19/USA/KY-CDC-2-4242084/2021) were used for the animal challenge studies and neutralization testing. SARS-CoV-2 Delta was obtained with contributions from B. Zhou, N. Thornburg, and S. Tong (Centers for Disease Control and Prevention, Atlanta, GA, USA). All viruses were grown and titered on VeroE6 cells, and sequences were confirmed.

### 2.4. Generation of VSV-Based Vaccine Candidates

VSVs expressing the SARS-CoV-2 S protein either alone (VSV-SARS2) or in combination with the EBOV GP (VSV-SARS2-EBOV) were used in this study. The generation of both vaccines, growth kinetics, and antigen expression have been previously described [33]. Briefly, for VSV-SARS2-EBOV, the SARS-CoV-2 S gene was PCR-amplified and cloned into the pATX-VSV-EBOV upstream of the EBOV GP. In order to support VSV-SARS2 replication, a cytoplasmic tail deletion was introduced into the S and the PCR product was cloned into pATX-VSV-EBOV, replacing the EBOV GP. The vaccine constructs were recovered as previously described [32].

### 2.5. RT-qPCR and Viral Titers

Oral swab samples were extracted using the QIAamp Viral RNA Mini Kit (Qiagen, Hilden, Germany) according to manufacturer specifications. Tissues, a maximum of 30 mg each, were processed and extracted using the RNeasy Mini Kit (Qiagen) according to manufacturer specifications. One-step RT-qPCR for genomic viral RNA was performed using specific primer–probe sets and the QuantiFast Probe RT-PCR +ROX Vial Kit (Qiagen) in the Rotor-Gene Q (Qiagen), as described previously [37]. Five μL of each RNA extract was run alongside dilutions of SARS-CoV-2 standards with a known concentration of RNA copies. 

SARS-CoV-2 titers were determined from lung samples using VeroE6 cells. Lung tissues were homogenized in 1 mL plain DMEM and cleared from debris by centrifugation. Then, 10-fold serial dilutions were generated in DMEM/2% FBS. Media was removed from confluent cells in 96-well plates and triplicates were inoculated with each dilution. Cells were monitored for cytopathic effect (CPE) and 50% tissue culture infectious dose (TCID_50_) was calculated for each sample. 

### 2.6. Enzyme-Linked Immunosorbent Assay

Serum samples from SARS-CoV-2-infected hamsters were inactivated by γ-irradiation and used in BSL2 according to IBC-approved SOPs. ELISA was performed using Nunc Maxisorp Immuno plates (Thermo Fisher Scientific) and recombinant SARS-CoV-2 S (S1 + S2) (Sino Biological, Chesterbrook, PA, USA) as previously described [34]. The OD values were normalized to the baseline samples obtained with naïve hamster serum and the cutoff value was set as the mean OD plus standard deviation of the blank.

### 2.7. Virus Neutralization Assay 

This assay was conducted on VeroE6 cells using three SARS-CoV-2 VOCs, as previously described [36]. CPE was documented, and the virus neutralization titer was expressed as the reciprocal value of the highest dilution of the serum which inhibited virus replication (no CPE).

### 2.8. Histology and Immunohistochemistry

Tissues were fixed in 10% neutral buffered formalin with two changes for a minimum of seven days. Tissues were processed as described previously [36]. All tissue slides were evaluated by a board-certified veterinary pathologist, and a 200× magnification photomicrograph of a representative hamster lung from each group was selected. 

### 2.9. Statistical Analyses

All statistical analysis was performed in Prism 9 (GraphPad, San Diego, CA, USA). A Mann–Whitney test was conducted to compare differences between groups for all data. Statistically significant differences are indicated as *p* < 0.0001 (****), *p* < 0.001 (***), *p* < 0.01 (**), and *p* < 0.05 (*).

## 3. Results

### 3.1. VSV-Based Vaccines Protect Hamsters from COVID-19 Caused by Three VOCs

**Figure 1:**
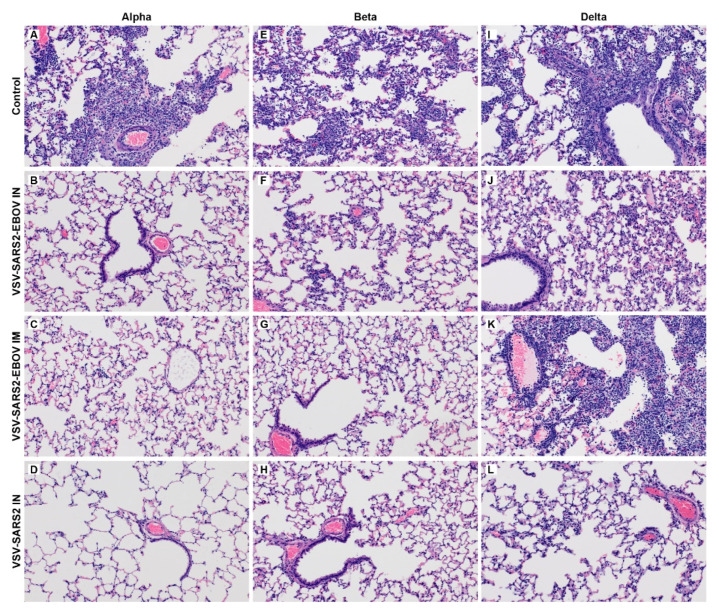
Histopathology of hamster lungs. Hamsters were vaccinated intramuscularly (IM) or intranasally (IN) 28 days prior to IN challenge with SARS-CoV-2 Alpha (**A**–**D**), Beta (**E**–**H**), and Delta (**I**–**L**) variants of concern (VOC). At 4 days post challenge (DPC), lung samples were collected and stained with H&E (200×). Moderate broncho-interstitial pneumonia and alveolar exudate was observed in all unvaccinated (control) hamsters regardless of the challenge variant (**A**,**E**,**I**). No (**B**,**D**) or minimal (**C**) interstitial pneumonia was observed in vaccinated, Alpha VOC-challenged hamsters. Minimal interstitial pneumonia was observed in vaccinated, Beta VOC-challenged hamsters (**F**–**H**). Minimal to mild interstitial pneumonia was observed in IN-vaccinated, Delta VOC-challenged hamsters (**J**,**L**). Histopathologic lesions were observed in IM-vaccinated, Delta VOC-challenged hamsters (**K**).

We sought to investigate the protective efficacy of our VSV-based COVID-19 vaccines with a longer time between vaccination and challenge to expand upon the previous study showing protective efficacy within 10 days [33]. We included the vaccines VSV-SARS2-EBOV, which is based on the licensed EBOV vaccine, and the VSV-SARS2 in our studies. Hamsters vaccinated with the parental VSV-EBOV served as a control group. Hamsters were vaccinated IM or IN with a single dose of either VSV-SARS2-EBOV or IN only with VSV-SARS2 (IM did not offer protection at 10 days [33]) 28 days prior to challenge with one of three SARS-CoV-2 VOC—the Alpha, Beta, or Delta variant. At 4 DPC, hamsters were euthanized to analyze lung pathology, viral load in oral swabs and lungs, and the magnitude and functionality of the humoral immune response. Primary evidence of protective efficacy for all three vaccination strategies tested was the lack of gross lung lesions apparent in the vaccinated animals compared to the control cohort of Alpha- or Beta-challenged hamsters (Figure S1). Hamsters challenged with the Delta variant were devoid of lesions when vaccinated IN but not IM (Figure S1). Histopathologic analysis revealed a lack of broncho-interstitial pneumonia and bronchiolitis for IN-vaccinated hamsters challenged with the Alpha variant (Figure 1B,D). The VSV-SARS2-EBOV IM-vaccinated hamsters presented with minimal interstitial pneumonia, but also displayed minimal cellular exudate and rare type II pneumocyte hyperplasia (Figure 1C). A more similar reduction of broncho-interstitial pneumonia was demonstrated in all vaccination groups in the hamsters challenged with the Beta variant (Figure 1F–H), with minimal interstitial pneumonia noted in all vaccination groups. Similar to the Alpha variant, the IN-vaccinated hamsters presented with the least amount of broncho-interstitial pneumonia when challenged with the Delta variant (Figure 1J,L). However, IM-vaccinated hamsters presented with more severe broncho-interstitial pneumonia and more robust cellular exudate (Figure 1K). Control hamsters presented with interstitial pneumonia after infection with all three VOC (Figure 1A,E,I).

**Figure 2:**
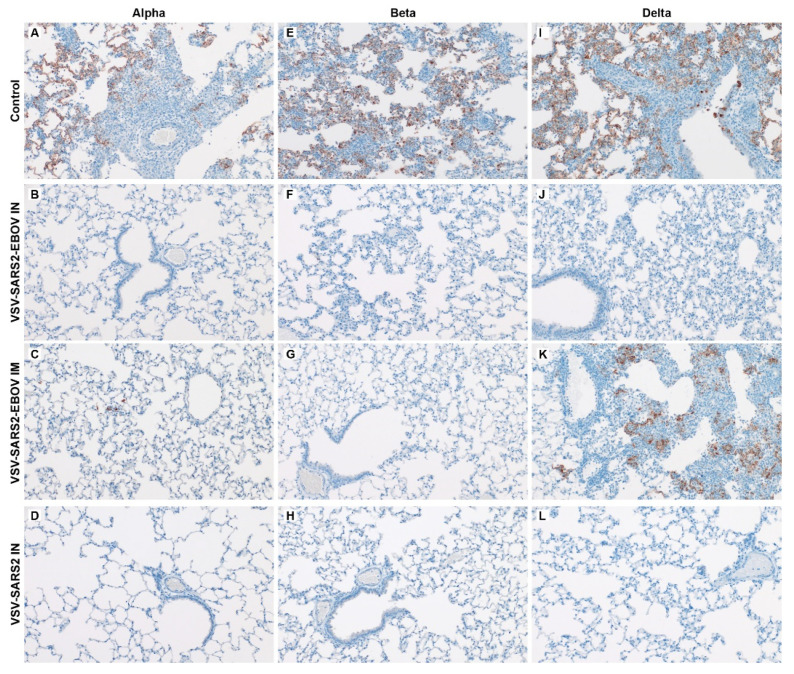
Immunohistochemistry of hamster lungs. Hamsters were vaccinated IM or IN 28 days prior to IN challenge with SARS-CoV-2 Alpha (**A**–**D**), Beta (**E**–**H**), and Delta (**I**–**L**) VOC. At 4 DPC lung samples were collected and stained with anti-SARS-CoV-2 nucleocapsid antibody (200×). Antigen immunoreactivity was readily detected in all control hamsters regardless of the challenge variant (**A**,**E**,**I**). Antigens were not detected in any VSV-SARS2-EBOV or VSV-SARS2 IN-vaccinated hamster regardless of challenge variant (**B**,**D**,**F**,**H**,**J**,**L**). Antigen immunoreactivity was observed only in pulmonary macrophages of VSV-SARS2-EBOV IM-vaccinated hamsters challenged with the Alpha VOC (**C**). No immunoreactivity was observed in VSV-SARS2-EBOV IM-vaccinated, Beta VOC-challenged hamsters (**G**). Abundant immunoreactivity was observed in VSV-SARS2-EBOV IM-vaccinated, Delta VOC-challenged hamsters (**K**).

Immunohistochemistry demonstrated that there was no immunoreactivity in hamsters IN-vaccinated and challenged with the Alpha variant (Figure 2B,C), while hamsters IM-vaccinated had antigen detection only in alveolar macrophages (Figure 2C). While minimal to mild pulmonary lesions were observed in all vaccinated hamsters when challenged with the Beta variant, no immunoreactivity was observed either in association with the lesions or in unaffected tissue sections, regardless of vaccination (Figure 2F–H). Reflective of the gross and histopathology determination, VSV-SARS2-EBOV IM vaccination showed increased antigen detection (Figure 2K) compared to the IN-vaccinated cohorts when challenged with the Delta variant (Figure 2J,L). The control vaccinated hamsters showed robust immunoreactivity in the bronchiolar epithelium, pneumocytes, and alveolar macrophages for all three VOC (Figure 2A,E,I).

### 3.2. VSV Vaccination Reduces Oral Shedding and Lung Viral Load

**Figure 3:**
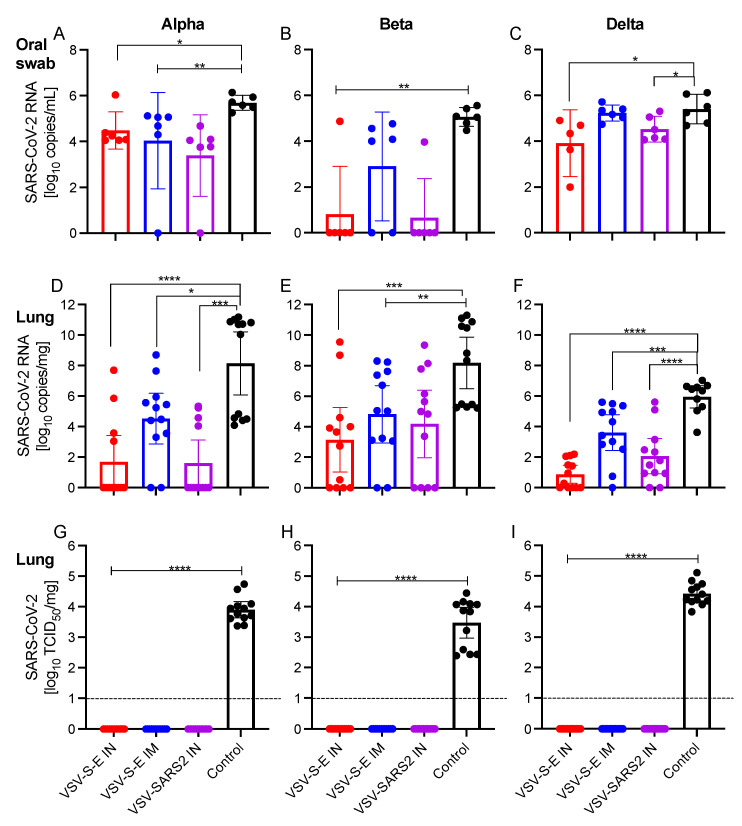
Vaccination reduces viral burden in hamsters. Hamsters were vaccinated IM or IN with VSV-SARS2-EBOV (VSV-S-E) or IN with VSV-SARS2 28 days prior to challenge with SARS-CoV-2 Alpha, Beta, or Delta VOC. At 4 DPC, oral swab and lung samples were collected. Levels of SARS-CoV-2 RNA in (**A**–**C**) oral swabs and (**D**–**F**) lung samples. (**G**–**I**) Virus titer in hamster lungs determined as median tissue culture infectious dose (TCID_50_). Geometric mean and geometric SD are depicted. Statistical significance as determined by the Mann–Whitney test is indicated as *p* < 0.0001 (****), *p* < 0.001 (***), *p* < 0.01 (**), and *p* < 0.05 (*).

The two key goals of vaccination against SARS-CoV-2 are to control viral replication in the lungs, and to reduce the risk of viral shedding that drives further virus transmission. We sought to investigate the ability of our vaccines to reduce viral load in the oral swabs and lungs of challenged hamsters. We found that all vaccines significantly reduced the amount of viral RNA in the oral swabs of vaccinated hamsters compared to the control cohort when challenged with the Alpha and Beta variants (Figure 3A,B). In contrast, only the IN-vaccinated hamsters had significantly reduced viral RNA in the oral swabs when challenged with the Delta variant (Figure 3C). Next, we investigated the level of control of viral replication in the lungs of these hamsters and found that all vaccines significantly reduced viral RNA in the lungs of vaccinated hamsters against all tested VOC (Figure 3D–F). We also determined the infectious virus titer within the lungs of all hamsters and found that any vaccination resulted in undetectable amounts of infectious virus in the lungs of vaccinated hamsters regardless of VOC challenge (Figure 3G–I). 

### 3.3. The Magnitude and Functionality of the Humoral Response Following VSV Vaccination

**Figure 4:**
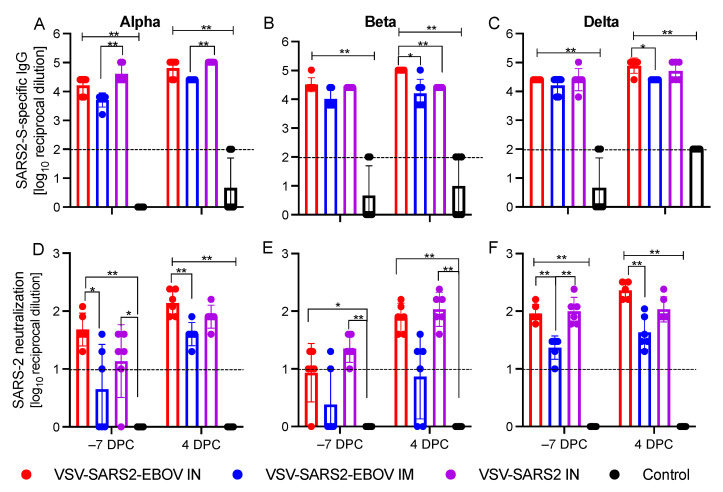
Humoral responses after vaccination and challenge. Hamsters were vaccinated IM or IN with VSV-SARS2-EBOV or IN with VSV-SARS2 28 days prior to challenge with SARS-CoV-2 Alpha, Beta, and Delta VOC. At −7 and 4 DPC serum samples were collected. (**A**–**C**) Levels of spike (S)-specific IgG were assessed. (**D**–**F**) The neutralization capacity against homologous viruses. Statistical significance as determined by the Mann–Whitney test is indicated as *p* < 0.01 (**) and *p* < 0.05 (*).

It is challenging to extensively analyze immunological responses following vaccination and infection in the hamster model, as there are limited reagents available. However, the antigen-specific antibody response and neutralization capacity are areas that can be readily investigated to gain insight into possible correlates of protection. We measured the S-specific humoral response 7 days prior to challenge (−7 DPC) and at the time of euthanisa (4 DPC). We determined a significantly higher amount of S-specific IgG at −7 DPC and at the time of euthanasia for all vaccinated hamsters compared to the control cohort regardless of the VOC used for challenge (Figure 4A–C). The S-specific IgG titer was significantly higher at both time points for VSV-SARS2 IN-vaccinated hamsters compared to the VSV-SARS2-EBOV IM-vaccinated group challenged with the Alpha variant (Figure 4A). We found that VSV-SARS2-EBOV IN-vaccinated hamsters had significantly higher S-pecific IgG titers at 4 DPC compared to the IM-vacciniated groups in the animals challenged with Beta and Delta variants (Figure 4B,C). Additionally, the VSV-SARS2-EBOV IN group had a higher S-specific IgG titer at 4 DPC compared to the VSV-SARS2 IN group challenged with the Beta variant (Figure 4B). To determine the functionality of this antigen-specific response, we employed a microneutralization assay using live virus in a matched approach (e.g., Alpha-challenged hamster serum sample neutralization with Alpha VOC) to ascertain neutralization titers. We found that IN vaccination with VSV-SARS2 and VSV-SARS2-EBOV resulted in a more robust functional response against all three VOC compared to the VSV-SARS2-EBOV IM group (Figure 4D–F). At −7 DPC, neutralization of the Delta variant was highest for all vaccinated hamsters (Figure 4F). In addition, all IN-vaccinated hamsters had significantly higher neutralizing titers at −7 DPC compared to the IM-vaccinated group against the Delta variant. This difference was maintained at the time of euthanasia for the VSV-SARS2-EBOV IN group compared to the IM-vaccinated cohort (Figure 4F).

## 4. Discussion

For effective control of the COVID-19 pandemic, vaccine-mediated protective immunity should not only prevent viral replication in the lower respiratory tract, it should also reduce or prevent SARS-CoV-2 shedding to eliminate viral transmission among humans [38,39]. The risk of asymptomatic viral spread is a direct consequence of the inability of current vaccines to prevent, at a minimum, upper respiratory tract viral shedding. In this study, we demonstrated that a VSV-based vaccine with a single dose administered 28 days prior to SARS-CoV-2 exposure is sufficient to protect hamsters from challenge with multiple VOC. Our results show decreased lung viral RNA levels and undetectable infectious viral titers of all vaccinated hamsters correlating with reduced or absent histopathologic lesions in the lower respiratory tree. We went on to show significant reductions in viral RNA in oral swabs of all vaccinated hamsters, regardless of vaccination route when challenged with the Alpha or Beta variants. IN vaccination regardless of the vaccine resulted in significant reduction of viral RNA in the oral swabs of Delta-challenged hamsters, consistent with previous findings in which IN vaccination was the most efficacious in a 10-day time to challenge study [33]. Taken together, these data suggest that a VSV-based vaccine successfully prevents severe lower respiratory disease associated with SARS-CoV-2 VOC infection and, importantly, may significantly reduce viral spread in the acute phase of infection. However, transmission studies will need to be conducted to confirm this hypothesis. 

The mutagenic nature of SARS-CoV-2 is a key driver of the continuing COVID-19 pandemic with iterative emergence of VOC that then become the dominant circulating strains globally. Determining the breadth of vaccine efficacy across emerging VOC and the optimal route of vaccination is needed to elicit the most protective immune response. Merck developed a VSV-SARS2 vaccine similar to ours but discontinued production after a Phase I clinical trial demonstrated a lower humoral response than after natural infection when IM-administered [40]. Our previous work and this work demonstrate that IN administration is the superior route of vaccination [26]. The two primarily distributed vaccines in the United States, Pfizer (BNT162b2) and Moderna (mRNA-1273), have demonstrated variable results in live virus neutralization tests against SARS-CoV-2 VOC. The neutralization potential against the Alpha variant remained similar to that of the ancestral strain but dropped significantly against the Beta variant—a 12.4- to 22.8-fold decrease for Moderna and Pfizer, respectively [15]. A similar trend has been demonstrated for the AstraZeneca ChAdOx1 nCOV/AZD1222 vaccine, with only 10.4–22% efficacy against the Beta variant [41,42,43]. The effectiveness of the Pfizer and Moderna vaccines against the Delta variant demonstrates the ability to stop severe disease with 93.4 and 96.1% efficacy, respectively. However, the ability to inhibit infection is much lower at 51.9% and 73.1% for Pfizer and Moderna, respectively [44]. A metanalysis study determined that the average fold decrease of live virus neutralization activity against the Delta variant for mRNA vaccines was 3.2-fold [16]. Due to waning immunity, booster vaccination doses have been encouraged by the CDC in the USA which allowed for the recovery of the humoral response magnitude. However, with the emergence of the newest VOC, Omicron, the efficacy of the booster vaccination doses have again been challenged. While the cross-reactive cellular response is maintained in the majority of individuals, the functionality of the cross-reactive antibody response declines dramatically [45,46,47,48]. We have previously demonstrated vaccine efficacy similar to the results presented here, inhibiting infection of the lungs and no effect on oral viral shedding [33]. This study highlights both of those attributes when the single vaccination was administered a longer time before challenge. Vaccine efficacy was retained in the lungs of challenged hamsters and the amount of viral shedding was significantly decreased, thus reducing both disease progression and potential risk of viral transmission. 

The production of a protective mucosal immune response is paramount for preventing SARS-CoV-2 transmission [38,49]. There are several challenges to overcome in preventing upper respiratory infection. First, after viral entry, ciliated nasal epithelial cells dramatically increase the expression of ACE2 and TMPRSS2, both of which facilitate viral entry and allow for more effective cell–cell propagation [50]. Second, a rapid burst of SARS-CoV-2 replication early in infection in the upper respiratory tract may overwhelm mucosal immunity stimulated [51,52]. Third, the functional systemic antibody response is less permissive to the upper respiratory tract, making it more difficult to protect this anatomic region. Due to these three major concerns we sought to use the IN vaccination route to allow for the highest likelihood of mucosal immunity generation. Through this study and our previous work, it became apparent that IN vaccination with our VSV constructs is the most efficient route of vaccination in the COVID-19 hamster model. We hypothesize that this is largely due to the generation of mucosal immunity. A caveat to both of our studies is the limited availability of immunological tools for hamsters at this time, particularly the ability to measure antigen-specific IgA response and to characterize functional cellular responses via flow cytometry. 

The VSV vaccine platform has multiple characteristics that contribute to its safety profile, which is an important consideration for a newly developed vaccine candidate. First, VSV is sensitive to interferon α/β, and an intact innate immune system can control VSV replication [25]. Additionally, we have replaced the VSV GP, which is considered the primary virulence factor, with that of SARS-CoV-2 S or EBOV GP [53]. Finally, the VSV-SARS2-EBOV vector was constructed utilizing the FDA- and EMA-approved EBOV vaccine by Merck (Ervebo) and further attenuated by the addition of the S antigen. We hypothesize that the addition of the EBOV GP promotes ACE2-independent infection and, therefore, would support increased viral replication, which we have previously demonstrated [33].

In summary, we generated two effective, single-dose vaccines against SARS-CoV-2 VOC. VSV-SARS2-EBOV is effective against the Alpha, Beta, and Delta variants when administered IN and was effective in diminishing lung lesion development as well as viral shedding, as indicated by the reduction of viral RNA levels in the oral swabs. Our results again suggest that IN is the optimal route of vaccination in the hamster model for VSV-based vaccines as well as other vaccines to stimulate a stronger immune response at the site of highest viral replication [54,55,56]. At this time, the VSV vaccines presented here have potential as an alternative boosting vaccine against COVID-19. The fast-acting potential and stimulation of primarily innate and humoral immune responses by these vaccines would complement the established immune response achieved by the COVID-19 adenovirus- and mRNA-based vaccinations. 

## Data Availability

The data presented in this study are available on request from the corresponding author.

## Supplementary Materials

The following are available online at https://www.mdpi.com/article/10.3390/vaccines10030435/s1. Figure S1. Hamster lung gross pathology.

## References

[1] Qiu X., Nergiz A.I., Maraolo A.E., Bogoch I.I., Low N., Cevik M. (2021). The role of asymptomatic and pre-symptomatic infection in SARS-CoV-2 transmission—A living systematic review. Clin. Microbiol. Infect..

[2] Goldberg Y., Mandel M., Bar-On Y.M., Bodenheimer O., Freedman L., Haas E.J., Milo R., Alroy-Preis S., Ash N., Huppert A. (2021). Waning Immunity after the BNT162b2 Vaccine in Israel. N. Engl. J. Med..

[3] Mizrahi B., Lotan R., Kalkstein N., Peretz A., Perez G., Ben-Tov A., Chodick G., Gazit S., Patalon T. (2021). Correlation of SARS-CoV-2-breakthrough infections to time-from-vaccine. Nat. Commun..

[4] Post N., Eddy D., Huntley C., van Schalkwyk M.C.I., Shrotri M., Leeman D., Rigby S., Williams S.V., Bermingham W.H., Kellam P. (2020). Antibody response to SARS-CoV-2 infection in humans: A systematic review. PLoS ONE.

[5] Shrotri M., van Schalkwyk M.C.I., Post N., Eddy D., Huntley C., Leeman D., Rigby S., Williams S.V., Bermingham W.H., Kellam P. (2021). T cell response to SARS-CoV-2 infection in humans: A systematic review. PLoS ONE.

[6] Faria N.R., Mellan T.A., Whittaker C., Claro I.M., Candido D.D.S., Mishra S., Crispim M.A.E., Sales F.C.S., Hawryluk I., McCrone J.T. (2021). Genomics and epidemiology of the P.1 SARS-CoV-2 lineage in Manaus, Brazil. Science.

[7] Chen R.E., Zhang X., Case J.B., Winkler E.S., Liu Y., VanBlargan L.A., Liu J., Errico J.M., Xie X., Suryadevara N. (2021). Resistance of SARS-CoV-2 variants to neutralization by monoclonal and serum-derived polyclonal antibodies. Nat. Med..

[8] Liu Y., Liu J., Xia H., Zhang X., Fontes-Garfias C.R., Swanson K.A., Cai H., Sarkar R., Chen W., Cutler M. (2021). Neutralizing Activity of BNT162b2-Elicited Serum. N. Engl. J. Med..

[9] Wibmer C.K., Ayres F., Hermanus T., Madzivhandila M., Kgagudi P., Oosthuysen B., Lambson B.E., De Oliveira T., Vermeulen M., Van der Berg K. (2021). SARS-CoV-2 501Y.V2 Escapes Neutralization by South African COVID-19 Donor Plasma. Nat. Med..

[10] Tian D., Sun Y., Zhou J., Ye Q. (2021). The Global Epidemic of the SARS-CoV-2 Delta Variant, Key Spike Mutations and Immune Escape. Front. Immunol..

[11] Mathieu E., Ritchie H., Ortiz-Ospina E., Roser M., Hasell J., Appel C., Giattino C., Rodés-Guirao L. (2021). A global database of COVID-19 vaccinations. Nat. Hum. Behav..

[12] Haas E.J., Angulo F.J., McLaughlin J.M., Anis E., Singer S.R., Khan F., Brooks N., Smaja M., Mircus G., Pan K. (2021). Impact and effectiveness of mRNA BNT162b2 vaccine against SARS-CoV-2 infections and COVID-19 cases, hospitalisations, and deaths following a nationwide vaccination campaign in Israel: An observational study using national surveillance data. Lancet.

[13] Dagan N., Barda N., Kepten E., Miron O., Perchik S., Katz M.A., Hernán M.A., Lipsitch M., Reis B., Balicer R.D. (2021). BNT162b2 mRNA Covid-19 Vaccine in a Nationwide Mass Vaccination Setting. N. Engl. J. Med..

[14] Noh J.Y., Jeong H.W., Shin E.-C. (2021). SARS-CoV-2 mutations, vaccines, and immunity: Implication of variants of concern. Signal Transduct. Target. Ther..

[15] Noori M., Nejadghaderi S.A., Arshi S., Carson-Chahhoud K., Ansarin K., Kolahi A., Safiri S. (2021). Potency of BNT162b2 and mRNA-1273 vaccine-induced neutralizing antibodies against severe acute respiratory syndrome-CoV-2 variants of concern: A systematic review of in vitro studies. Rev. Med. Virol..

[16] Chen X., Chen Z., Azman A.S., Sun R., Lu W., Zheng N., Zhou J., Wu Q., Deng X., Zhao Z. (2021). Neutralizing antibodies against SARS-CoV-2 variants induced by natural infection or vaccination: A systematic review and pooled meta-analysis. Clin. Infect. Dis..

[17] Zhang M., Liang Y., Yu D., Du B., Cheng W., Li L., Yu Z., Luo S., Zhang Y., Wang H. (2022). A systematic review of Vaccine Breakthrough Infections by SARS-CoV-2 Delta Variant. Int. J. Biol. Sci..

[18] Tartof S.Y., Slezak J.M., Fischer H., Hong V., Ackerson B.K., Ranasinghe O.N., Frankland T.B., Ogun O.A., Zamparo J.M., Gray S. (2021). Effectiveness of mRNA BNT162b2 COVID-19 vaccine up to 6 months in a large integrated health system in the USA: A retrospective cohort study. Lancet.

[19] Hoffmann M., Krüger N., Schulz S., Cossmann A., Rocha C., Kempf A., Nehlmeier I., Graichen L., Moldenhauer A.-S., Winkler M.S. (2022). The Omicron variant is highly resistant against antibody-mediated neutralization: Implications for control of the COVID-19 pandemic. Cell.

[20] Kim Y.-K., Minn D., Chang S.-H., Suh J.-S. (2022). Comparing SARS-CoV-2 Antibody Responses after Various COVID-19 Vaccinations in Healthcare Workers. Vaccines.

[21] Ponce-de-Leon S., Torres M., Soto-Ramirez L.E., Jose Calva J., Santillan-Doherty P., Carranza-Salazar D.E., Carreno J.M., Carranza C., Juarez E., Carreto-Binaghi L.E. (2022). Safety and immunogenicity of a live recombinant Newcastle disease virus-based COVID-19 vaccine (Patria) administered via the intramuscular or intranasal route: Interim results of a non-randomized open label phase I trial in Mexico. medRxiv.

[22] Sun W., Liu Y., Amanat F., González-Domínguez I., McCroskery S., Slamanig S., Coughlan L., Rosado V., Lemus N., Jangra S. (2021). A Newcastle disease virus expressing a stabilized spike protein of SARS-CoV-2 induces protective immune responses. Nat. Commun..

[23] Marzi A., Robertson S.J., Haddock E., Feldmann F., Hanley P.W., Scott D.P., Strong J.E., Kobinger G.P., Best S.M., Feldmann H. (2015). VSV-EBOV rapidly protects macaques against infection with the 2014/15 Ebola virus outbreak strain. Science.

[24] Marzi A., Feldmann H., Geisbert T.W., Falzarano D. (2011). Vesicular Stomatitis Virus-Based Vaccines for Prophylaxis and Treatment of Filovirus Infections. J. Bioterror. Biodef..

[25] Fathi A., Dahlke C., Addo M. (2019). Recombinant vesicular stomatitis virus vector vaccines for WHO blueprint priority pathogens. Hum. Vaccines Immunother..

[26] Fultz P.N., Holland J.J. (1985). Differing responses of hamsters to infection by vesicular stomatitis virus Indiana and New Jersey serotypes. Virus Res..

[27] Fultz P.N., Shadduck J.A., Kang C.Y., Streilein J.W. (1981). Genetic analysis of resistance to lethal infections of vesicular stomatitis virus in Syrian hamsters. Infect. Immun..

[28] Brown K.S., Safronetz D., Marzi A., Ebihara H., Feldmann H. (2011). Vesicular Stomatitis Virus-Based Vaccine Protects Hamsters against Lethal Challenge with Andes Virus. J. Virol..

[29] Furuyama W., Reynolds P., Haddock E., Meade-White K., Le M.Q., Kawaoka Y., Feldmann H., Marzi A. (2020). A single dose of a vesicular stomatitis virus-based influenza vaccine confers rapid protection against H5 viruses from different clades. NPJ Vaccines.

[30] Marzi A., Menicucci A.R., Engelmann F., Callison J., Horne E.J., Feldmann F., Jankeel A., Feldmann H., Messaoudi I. (2020). Protection Against Marburg Virus Using a Recombinant VSV-Vaccine Depends on T and B Cell Activation. Front. Immunol..

[31] Safronetz D., Mire C., Rosenke K., Feldmann F., Haddock E., Geisbert T., Feldmann H. (2015). A Recombinant Vesicular Stomatitis Virus-Based Lassa Fever Vaccine Protects Guinea Pigs and Macaques against Challenge with Geographically and Genetically Distinct Lassa Viruses. PLoS Negl. Trop. Dis..

[32] Emanuel J., Callison J., Dowd K.A., Pierson T.C., Feldmann H., Marzi A. (2018). A VSV-based Zika virus vaccine protects mice from lethal challenge. Sci. Rep..

[33] O’Donnell K.L., Clancy C.S., Griffin A.J., Shifflett K., Gourdine T., Thomas T., Long C.M., Furuyama W., Marzi A. (2022). Optimization of Single-Dose VSV-Based COVID-19 Vaccination in Hamsters. Front. Immunol..

[34] Furuyama W., Shifflett K., Pinski A.N., Griffin A.J., Feldmann F., Okumura A., Gourdine T., Jankeel A., Lovaglio J., Hanley P.W. (2022). Rapid Protection from COVID-19 in Nonhuman Primates Vaccinated Intramuscularly but Not Intranasally with a Single Dose of a Vesicular Stomatitis Virus-Based Vaccine. mBio.

[35] Rosenke K., Meade-White K., Letko M., Clancy C., Hansen F., Liu Y., Okumura A., Tang-Huau T.-L., Li R., Saturday G. (2020). Defining the Syrian hamster as a highly susceptible preclinical model for SARS-CoV-2 infection. Emerg. Microbes Infect..

[36] O’Donnell K.L., Pinski A.N., Clancy C.S., Gourdine T., Shifflett K., Fletcher P., Messaoudi I., Marzi A. (2021). Pathogenic and transcriptomic differences of emerging SARS-CoV-2 variants in the Syrian golden hamster model. eBioMedicine.

[37] Van Doremalen N., Lambe T., Spencer A., Belij-Rammerstorfer S., Purushotham J.N., Port J.R., Avanzato V.A., Bushmaker T., Flaxman A., Ulaszewska M. (2020). ChAdOx1 nCoV-19 vaccine prevents SARS-CoV-2 pneumonia in rhesus macaques. Nature.

[38] Krammer F. (2020). SARS-CoV-2 vaccines in development. Nature.

[39] Baden L.R., El Sahly H.M., Essink B., Kotloff K., Frey S., Novak R., Diemert D., Spector S.A., Rouphael N., Creech C.B. (2021). Efficacy and Safety of the mRNA-1273 SARS-CoV-2 Vaccine. N. Engl. J. Med..

[40] Merck & Co., Inc. (2021). Merck Discontinues Development of SARS-CoV-2/COVID-19 Vaccine Candidates; Continues Development of Two Investigational Therapeutic Candidates. https://www.merck.com/news/merck-discontinues-development-of-sars-cov-2-covid-19-vaccine-candidates-continues-development-of-two-investigational-therapeutic-candidates/.

[41] Harvey W.T., Carabelli A.M., Jackson B., Gupta R.K., Thomson E.C., Harrison E.M., Ludden C., Reeve R., Rambaut A., Consortium C.-G.U. (2021). SARS-CoV-2 variants, spike mutations and immune escape. Nat. Rev. Microbiol..

[42] Knoll M.D., Wonodi C. (2021). Oxford-AstraZeneca COVID-19 vaccine efficacy. Lancet.

[43] Madhi S.A., Baillie V., Cutland C.L., Voysey M., Koen A.L., Fairlie L., Padayachee S.D., Dheda K., Barnabas S.L., Bhorat Q.E. (2021). Efficacy of the ChAdOx1 nCoV-19 Covid-19 Vaccine against the B.1.351 Variant. N. Engl. J. Med..

[44] Tang P., Hasan M.R., Chemaitelly H., Yassine H.M., Benslimane F.M., Al Khatib H.A., AlMukdad S., Coyle P., Ayoub H.H., Al Kanaani Z. (2021). BNT162b2 and mRNA-1273 COVID-19 vaccine effectiveness against the SARS-CoV-2 Delta variant in Qatar. Nat. Med..

[45] Khong K.-W., Liu D., Leung K.-Y., Lu L., Lam H.-Y., Chen L., Chan P.-C., Lam H.-M., Xie X., Zhang R. (2022). Antibody Response of Combination of BNT162b2 and CoronaVac Platforms of COVID-19 Vaccines against Omicron Variant. Vaccines.

[46] Kuhlmann C., Mayer C.K., Claassen M., Maponga T., Burgers W.A., Keeton R., Riou C., Sutherland A.D., Suliman T., Shaw M.L. (2022). Breakthrough infections with SARS-CoV-2 omicron despite mRNA vaccine booster dose. Lancet.

[47] Loconsole D., Bisceglia L., Centrone F., Sallustio A., Accogli M., Dalfino L., Brienza N., Chironna M. (2022). Autochthonous Outbreak of SARS-CoV-2 Omicron Variant in Booster-Vaccinated (3 Doses) Healthcare Workers in Southern Italy: Just the Tip of the Iceberg?. Vaccines.

[48] Naranbhai V., Nathan A., Kaseke C., Berrios C., Khatri A., Choi S., Getz M.A., Tano-Menka R., Ofoman O., Gayton A. (2022). T cell reactivity to the SARS-CoV-2 Omicron variant is preserved in most but not all individuals. Cell.

[49] Hassan A.O., Kafai N.M., Dmitriev I.P., Fox J.M., Smith B.K., Harvey I.B., Chen R.E., Winkler E.S., Wessel A.W., Case J.B. (2020). A Single-Dose Intranasal ChAd Vaccine Protects Upper and Lower Respiratory Tracts against SARS-CoV-2. Cell.

[50] Liu L., To K.K.-W., Chan K.-H., Wong Y.-C., Zhou R., Kwan K.-Y., Fong C.H.-Y., Chen L.-L., Choi C.Y.-K., Lu L. (2020). High neutralizing antibody titer in intensive care unit patients with COVID-19. Emerg. Microbes Infect..

[51] Peiris J.S.M., Chu C.M., Cheng V., Chan K., Hung I.F.N., Poon L., Law K., Tang B., Hon T., Chan C. (2003). Clinical progression and viral load in a community outbreak of coronavirus-associated SARS pneumonia: A prospective study. Lancet.

[52] Liu L., Wei Q., Nishiura K., Peng J., Wang H., Midkiff C.C., Alvarez X., Qin C., Lackner A., Chen Z. (2016). Spatiotemporal interplay of severe acute respiratory syndrome coronavirus and respiratory mucosal cells drives viral dissemination in rhesus macaques. Mucosal Immunol..

[53] Rose N.F., Roberts A., Buonocore L., Rose J.K. (2000). Glycoprotein Exchange Vectors Based on Vesicular Stomatitis Virus Allow Effective Boosting and Generation of Neutralizing Antibodies to a Primary Isolate of Human Immunodeficiency Virus Type 1. J. Virol..

[54] van Doremalen N., Purushotham J.N., Schulz J.E., Holbrook M.G., Bushmaker T., Carmody A., Port J.R., Yinda C.K., Okumura A., Saturday G. (2021). Intranasal ChAdOx1 nCoV-19/AZD1222 vaccination reduces viral shedding after SARS-CoV-2 D614G challenge in preclinical models. Sci. Transl. Med..

[55] An X., Martinez-Paniagua M., Rezvan A., Sefat S.R., Fathi M., Singh S., Biswas S., Pourpak M., Yee C., Liu X. (2021). Single-dose intranasal vaccination elicits systemic and mucosal immunity against SARS-CoV-2. iScience.

[56] King R., Silva-Sanchez A., Peel J., Botta D., Dickson A., Pinto A., Meza-Perez S., Allie S., Schultz M., Liu M. (2021). Single-Dose Intranasal Administration of AdCOVID Elicits Systemic and Mucosal Immunity against SARS-CoV-2 and Fully Protects Mice from Lethal Challenge. Vaccines.

