# Supplementary material for: VSV-Based Vaccines Reduce Virus Shedding and Viral Load in Hamsters Infected with SARS-CoV-2 Variants of Concern
Source: Vaccines (Basel). 2022 Mar 12;10(3):435. doi: 10.3390/vaccines10030435 (PMC8951568; doi:10.3390/vaccines10030435)
Supplement: Supplementary file 1 [file vaccines-10-00435-s001.zip › vaccines-1611998-supplementary.pdf]

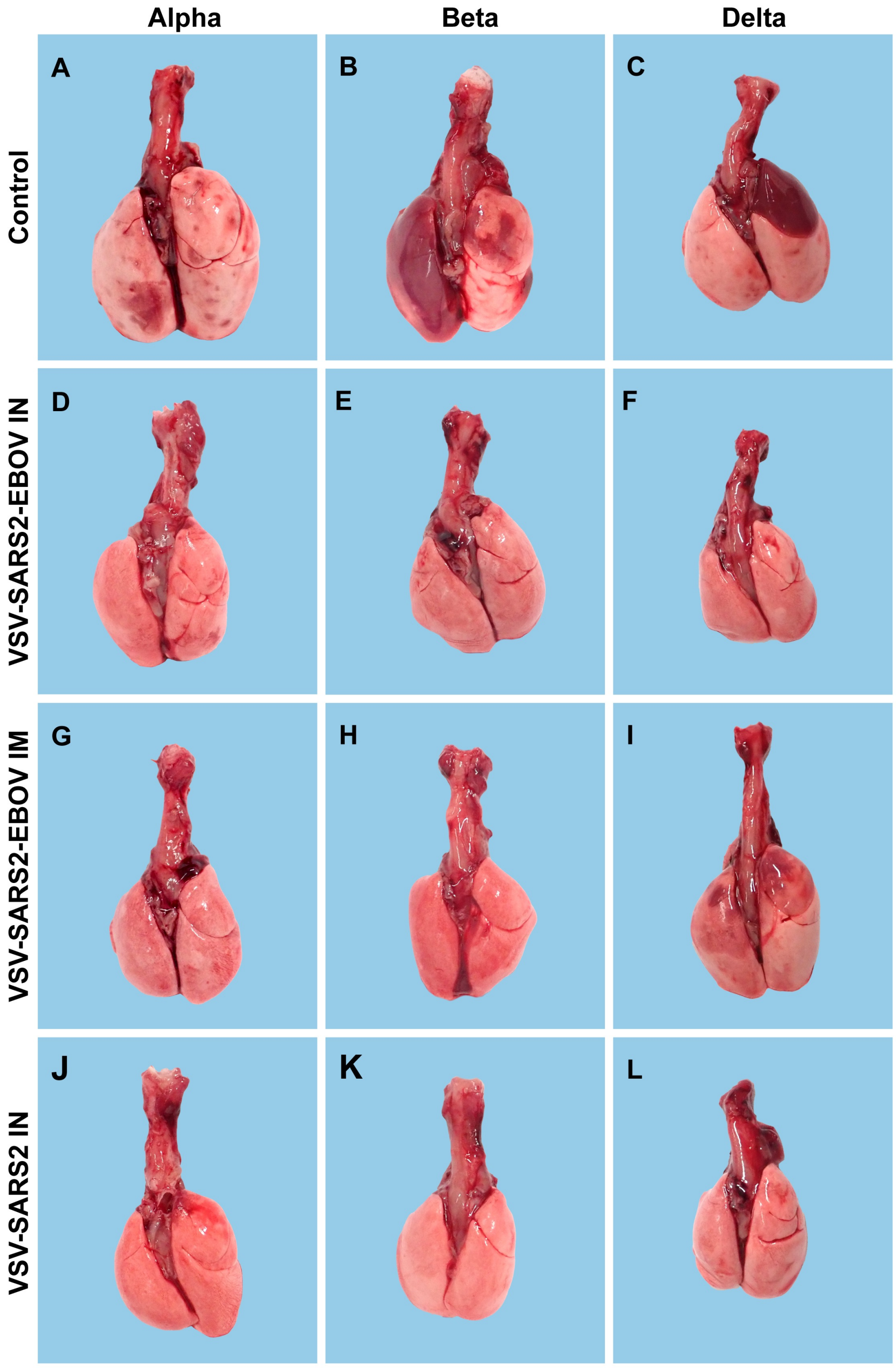

Figure S1. Hamster lung gross pathology. Vaccinated hamsters were challenged with SARS-CoV-2 alpha, beta, or delta VOC. At 4 days post challenge, hamsters were euthanized for sample collection. Lung gross pathology of a representative hamster in each group is depicted.
